# A Case Report of Iliac Punch-Out Grafting: A Novel Surgical Technique for Angled Bony Increased Offset Reverse Shoulder Arthroplasty

**DOI:** 10.7759/cureus.86176

**Published:** 2025-06-16

**Authors:** Kazuhiro Ikeda, Shotaro Teruya, Hiromitsu Tsuge, Shinzo Onishi

**Affiliations:** 1 Department of Orthopedic Surgery, Institute of Clinical Medicine, University of Tsukuba, Tsukuba, JPN; 2 Department of Orthopedic Surgery, Kikkoman General Hospital, Noda, JPN

**Keywords:** bony increased offset, complication, iliac crest bone grafting, reverse shoulder arthroplasty, surgical technique

## Abstract

Conventional iliac crest bone grafting is often used in shoulder reconstruction; however, it may lead to donor-site complications. To address this concern, we applied a novel punch-out technique that enables cylindrical bone harvesting from the iliac wing while preserving the iliac crest. A 61-year-old woman with long-standing rheumatoid arthritis presented with right shoulder pain and functional impairment. Imaging revealed severe glenoid bone loss with insufficient humeral bone stock for standard reconstruction. She underwent a bony increased offset reverse shoulder arthroplasty using iliac punch-out grafting. The procedure was completed without complications, and the postoperative course was favorable. This technique enabled bone grafting without compromising the iliac crest. In this case, no donor-site complications were observed, and the clinical outcome was favorable. Iliac punch-out grafting may therefore be considered a potential option for structural reconstruction in reverse shoulder arthroplasty, although further investigation is warranted.

## Introduction

Iliac bone grafting is a common technique used in orthopedic reconstructive surgery, particularly in reverse shoulder arthroplasty (RSA) for destructive shoulder conditions [1]. The iliac bone is highly suitable for grafting because it possesses all three essential properties required for bone formation: osteoconduction, osteoinduction, and osteogenesis [2]. In particular, structural grafts harvested from the iliac crest, comprising both cortical and cancellous components in a three-dimensional configuration, are valuable in cases requiring mechanical support at the recipient site [3,4].

However, harvesting from the iliac crest can occasionally lead to donor-site complications such as cosmetic deformity, chronic pain, iliac wing fractures, and abdominal wall incisional hernia [3-7]. We introduced a novel technique called "iliac punch-out grafting", which preserves the iliac crest to address these concerns. In this technique, a cylindrical graft is punched from the iliac wing using the same reamer designed for bony increased offset reverse shoulder arthroplasty (BIO-RSA) [8]. Here, we present a case in which this technique was applied to an angled BIO-RSA and discuss its technical feasibility, potential advantages, and clinical implications. To the best of our knowledge, this is the first documented use of this technique.

## Case presentation

Patient and clinical history

A 61-year-old right-handed woman presented with severe pain and limited mobility in the right shoulder. She was diagnosed with rheumatoid arthritis and was treated with salazosulfapyridine and iguratimod. Her height was 161 cm, weight 41 kg, and body mass index (BMI) 16 kg/m². The range of motion in the right shoulder was limited to 60° forward elevation and 15° external rotation (first position). The hand-to-back movements were restricted to the upper buttocks. Her preoperative American Shoulder and Elbow Surgeons (ASES) score was 13, indicating significant impairment in activities of daily living; scores above 90 are generally considered to reflect near-normal shoulder function [9].

**Figure 1:**
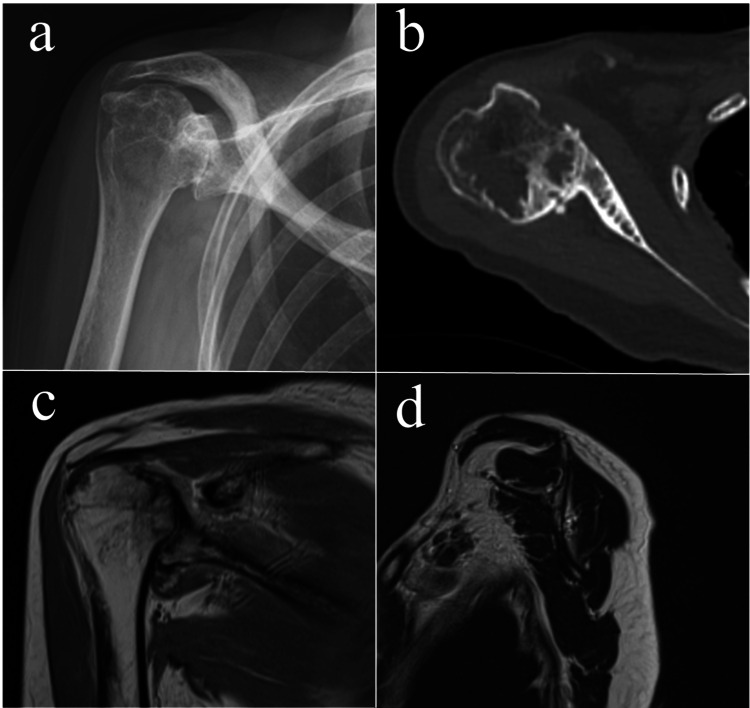
Preoperative imaging findings. (a) Plain radiograph (anteroposterior view). (b) Computed tomography (axial view). (c) Magnetic resonance imaging (T2-weighted image, coronal view). (d) Magnetic resonance imaging (T2-weighted image, sagittal view).

The preoperative imaging findings are shown in Figure 1. Plain radiographs revealed superior glenoid bone loss and collapse of the humeral head, which had migrated to the superior glenoid (Favard classification: Type E2 [10]). Computed tomography (CT) showed posterior glenoid bone loss with a glenoid retroversion angle of 32.8° (Walsh classification: Type B3 [11]) and a posterior subluxation ratio of 76.9% [12]; bone loss extended up to 10 mm at the superior and posterior aspects. A large bone cyst was also present in the humeral head. Magnetic resonance imaging revealed thinning of the rotator cuff but no apparent tears.

Based on these findings, the patient was diagnosed with destructive shoulder arthritis due to rheumatoid arthritis. Given this condition, we planned BIO-RSA, as poor native glenoid bone quality raised concerns about the fixation stability of a metal augment. The iliac bone was selected as the graft source as the humeral head lacked sufficient bone stock. Moreover, we anticipated that disruption of the iliac crest would pose a high risk of donor-site complications, including cosmetic deformities and pain, because the patient was markedly emaciated. Therefore, we introduced a novel bone-harvesting technique, iliac punch-out grafting, that enables the preservation of the iliac crest and planned to apply it in this case.

Operative procedure

This technique was applied as a modification of the iliac window harvesting method described by Behairy and Al-Sebai [13], which uses a rectangular opening. In the present case, a circular punch-out was performed using a cylindrical reamer. Prior to surgery, we confirmed sufficient bone thickness at the planned harvesting site using pelvic CT.

**Figure 2:**
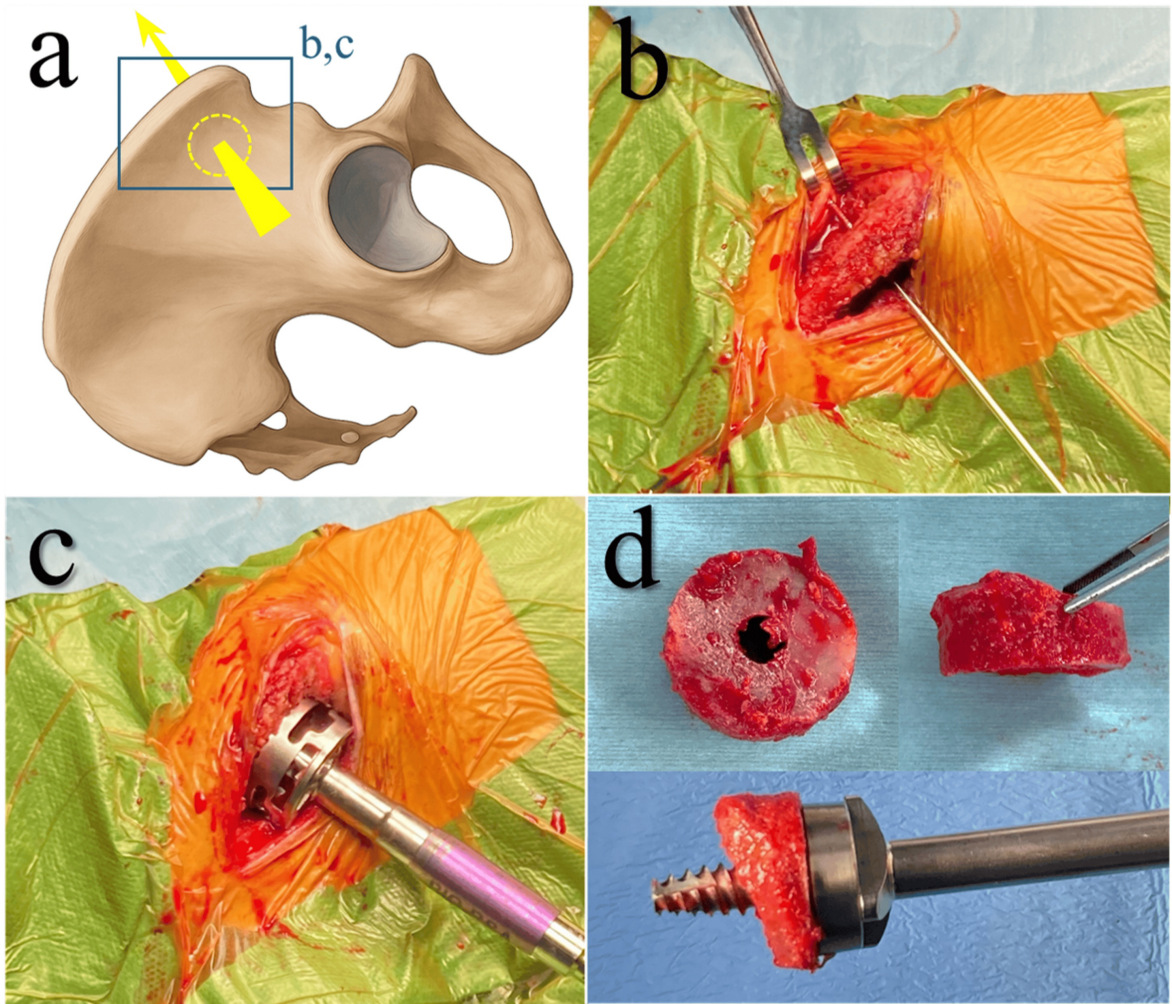
Iliac punch-out grafting. (a) Schematic illustration (Image Credits: Kazuhiro Ikeda). (b) Guide pin insertion into the iliac wing. (c) Punch-out of the iliac wing using a crown reamer. (d) Harvested cylindrical graft.

Iliac punch-out grafting was performed on the right iliac bone with the patient in the beach chair position (Figure 2). The harvesting site was selected as 2 cm posterior to the anterior superior iliac spine to preserve the mechanical strength of the iliac wing and avoid injury to the lateral femoral cutaneous nerve and the superior gluteal artery [14,15]; no intraoperative navigation or fluoroscopic guidance was used. The iliac crest was exposed, and the muscles attached to the inner and outer tables were gently elevated to expose the iliac wings at the planned site. A guide pin was inserted at the center of the harvesting area, and a cylindrical graft was punched out using a 29-mm crown reamer designed for BIO-RSA. The graft was shaped to a maximum thickness of 10 mm and a minimum of 6.5 mm, during which the cortical surface was lightly removed. The donor cavity was filled with β-tricalcium phosphate (β-TCP): two rectangular blocks (Affinos®, Kuraray Co., Ltd., Tokyo, Japan), each measuring 10×10×20 mm, were used to fill the defect. The blocks were relatively well stabilized within the cavity by being packed against the surrounding iliac muscle attachments.

Then, RSA was performed via a deltopectoral approach using the Aequalis Ascend Flex humeral stem and Aequalis Reversed II glenoid component (Stryker, Mahwah, New Jersey, United States). The graft was used to reconstruct the anterosuperior glenoid defect, and angled BIO-RSA was performed, targeting an RSA angle of 0° [16,17]. At the time of graft placement, two small holes were drilled into the native glenoid surface using a 2-mm drill to promote graft-to-host bone union by facilitating vascular and cellular ingrowth. The subscapularis tendon, which detached during exposure, was repaired at the end of the procedure. The total operative time was 137 minutes, and the estimated blood loss was 70 mL.

The arm was immobilized in an abduction brace for one week, and pendulum exercises were initiated. Lower extremity weight-bearing was permitted as soon as possible. Active elevation exercises, excluding external rotation, were performed from weeks 1 to 3. Range-of-motion exercises were performed without restriction after three weeks.

Postoperative course

**Figure 3:**
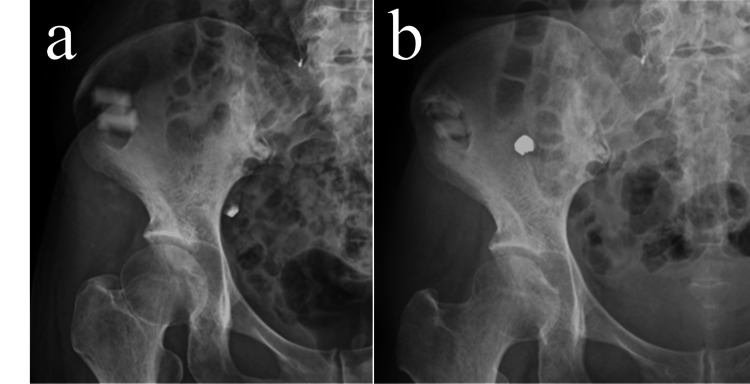
Plain radiographs of the donor site. (a) Immediately after surgery. (b) Two years postoperatively.

Postoperatively, the patient began assisted walking on day 2, progressed to independent walking by day 6, and was discharged on day 13. She had no complaints at the donor site, and her Majeed pelvic outcome score was 100 at the two-year follow-up, indicating full functional recovery without pelvic-related discomfort. Radiographs demonstrated a gradual radiographic appearance of bone replacement in the donor cavity (Figure 3).

**Figure 4:**
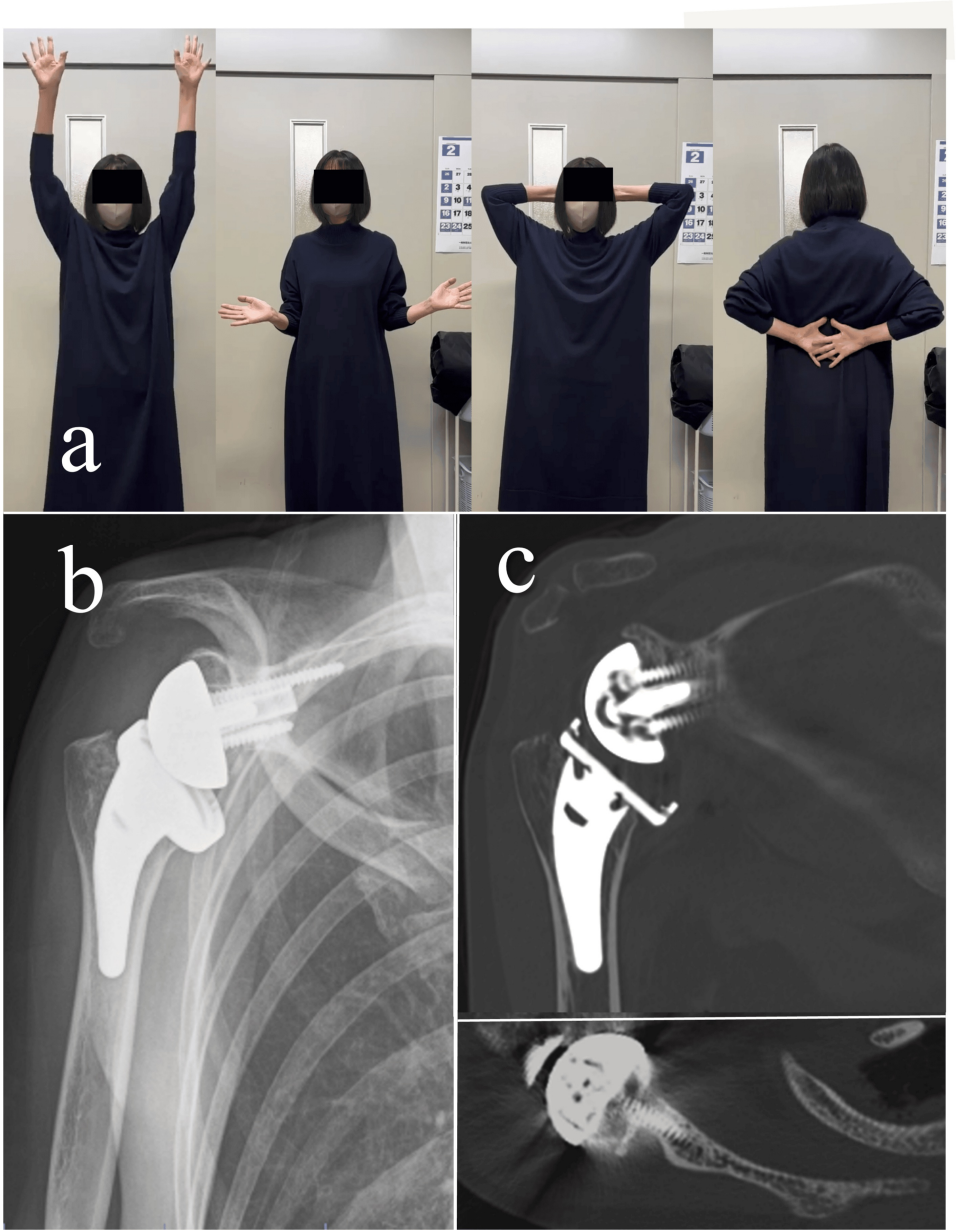
Postoperative course of the shoulder. (a) Shoulder range of motion two years postoperatively. (b) Plain radiograph immediately after surgery. (c) Computed tomography (coronal and axial views) two years postoperatively.

The shoulder range of motion was as follows at the two-year follow-up (Figure 4): forward elevation, 160°; abduction, 160°; external rotation (first position), 20°; external rotation (second position), 85°; and internal rotation to the L1 level. The patient reported no shoulder pain, with a numerical rating scale of 0. The ASES score improved to 95, suggesting almost full functional recovery. On plain radiographs, the RSA angle was 0°, and the lateralization shoulder angle [18] was 107°, indicating adequate glenoid inclination and substantial lateralization. CT revealed a complete graft union without evidence of implant loosening.

## Discussion

In this case, iliac punch-out grafting provided a structurally robust and appropriately sized bone graft for reconstructing a relatively large glenoid defect. Importantly, preservation of the iliac crest using this technique offers several clinical advantages.

First, preservation of the iliac crest maintains pelvic symmetry and reduces the risk of cosmetic deformities, particularly pronounced in thin or emaciated patients.

Second, and perhaps most importantly, preserving the iliac crest may reduce donor site pain, which has been reported in up to 34% of patients who undergo iliac bone grafting [4,7]. This pain is thought to be associated with localized mechanical irritation due to the irregularity of the iliac crest as well as increased mechanical stress on the iliac wing [7,15]. The iliac crest, which is continuous with the anterior superior and inferior iliac spines, must withstand tensile forces generated by the hip flexor muscles [14]. Accordingly, discontinuity of the iliac crest increases the stress on the iliac wing, potentially leading to motion-related pain or fractures [15]. Our technique preserves the structural continuity of the iliac crest, which may help maintain its mechanical function as a load-bearing region. Additionally, circular openings distribute stress more evenly than rhomboid windows [19]. Compared to the conventional rectangular window technique [13], our approach may offer biomechanical advantages; however, further testing is needed to confirm its relevance in iliac wing harvesting.

Third, this approach facilitates the closure of the donor site. Bone defects are typically surrounded by muscle, allowing for the stable packing of artificial bone. In the present case, the β-TCP placed in the cavity was radiographically replaced with autologous bone. Full-thickness harvesting of both the iliac crest and wing is associated with an increased risk of abdominal wall hernia [5]. In contrast, our technique enables effective closure of the defect, which may help prevent such complications [20].

As a result, the concept of cylindrical autologous bone harvesting may also be applicable to other procedures requiring structural bone grafts, such as spine, trauma, or joint reconstruction surgeries.

One limitation of iliac punch-out grafting is the limited graft thickness as the iliac bone gradually becomes thinner away from the crest. In contrast, the iliac crest harvesting technique allows controlled graft thickness based on the defect size. However, in our experience, the iliac crest graft may not provide sufficient width to accommodate the glenoid baseplate, particularly in smaller patients. Given these differences, the grafting technique should be based on the specific morphology of glenoid bone loss. In addition, while iliac punch-out grafting was safely performed in the beach chair position in the present thin patient, obese individuals may present technical challenges due to the thickness of subcutaneous tissue in the abdominal and gluteal regions, which can hinder exposure of the iliac wing. From the perspectives of postoperative cosmetic outcomes, reduced risk of local irritation, and technical accessibility, this technique may be particularly suitable for patients with low body mass.

## Conclusions

In this case, iliac punch-out grafting enabled the structural reconstruction of a large glenoid defect while preserving the continuity of the iliac crest. The technique was associated with favorable clinical outcomes and appeared safe with regard to donor site morbidity in this case. While further investigation is needed, this case suggests that iliac punch-out grafting could be considered as a surgical option in patients with substantial glenoid bone loss in whom autologous grafting from the humeral head is not feasible.
